# Differential Expression of Surface Markers in Mouse Bone Marrow Mesenchymal Stromal Cell Subpopulations with Distinct Lineage Commitment

**DOI:** 10.1371/journal.pone.0051221

**Published:** 2012-12-07

**Authors:** Maria Rostovskaya, Konstantinos Anastassiadis

**Affiliations:** BIOTEC, Technische Universitaet Dresden, Dresden, Germany; Georgia Health Sciences University, United States of America

## Abstract

Bone marrow mesenchymal stromal cells (BM MSCs) represent a heterogeneous population of progenitors with potential for generation of skeletal tissues. However the identity of BM MSC subpopulations is poorly defined mainly due to the absence of specific markers allowing *in situ* localization of those cells and isolation of pure cell types. Here, we aimed at characterization of surface markers in mouse BM MSCs and in their subsets with distinct differentiation potential. Using conditionally immortalized BM MSCs we performed a screening with 176 antibodies and high-throughput flow cytometry, and found 33 markers expressed in MSCs, and among them 3 were novel for MSCs and 13 have not been reported for MSCs from mice. Furthermore, we obtained clonally derived MSC subpopulations and identified bipotential progenitors capable for osteo- and adipogenic differentiation, as well as monopotential osteogenic and adipogenic clones, and thus confirmed heterogeneity of MSCs. We found that expression of CD200 was characteristic for the clones with osteogenic potential, whereas SSEA4 marked adipogenic progenitors lacking osteogenic capacity, and CD140a was expressed in adipogenic cells independently of their efficiency for osteogenesis. We confirmed our observations in cell sorting experiments and further investigated the expression of those markers during the course of differentiation. Thus, our findings provide to our knowledge the most comprehensive characterization of surface antigens expression in mouse BM MSCs to date, and suggest CD200, SSEA4 and CD140a as markers differentially expressed in distinct types of MSC progenitors.

## Introduction

Bone marrow stroma is a complex tissue consisting of many cell types, which provide a microenvironment for haematopoiesis and also contribute to the maintenance and regeneration of skeletal tissues [1], [2], [3]. Perturbed function of stromal tissue in humans can cause severe defects of skeletal system [4], [5], [6], [7] or dysregulation of haematopoiesis resulting in myelodysplasia and acute myeloid leukemia [8], [9]. On the other hand, capacity of stromal cells to differentiate into osteoblasts and chondrocytes makes them an important source for tissue engineering [10], [11] and provided success of treatment of patients with osteogenesis imperfecta [12], and immunosuppressive properties have already brought them to application in clinic to suppress graft-versus-host disease, GVHD [13].

These facts underline a need for developing protocols for cell replacement therapies, improving methods for stromal cell isolation and also understanding a physiological role of cellular components of bone marrow stroma.

Life-term replacement of stromal cells is supported by self-renewing mesenchymal stem cells that possess several characteristics including (1) clonogenicity *in vitro*, (2) multilineage differentiation into the cells of mesenchymal origin, including osteogenic, adipogenic and chondrogenic, and (3) ability to establish haematopoiesis upon transplantation. However, current methods of stromal cell isolation do not allow derivation of a pure population of stem cells and therefore cultured bone marrow stromal cells represent a mixture of their descendants, including progenitors of different types, hereafter referred as mesenchymal stromal cells (MSCs).

The isolation of pure cell types from bone marrow stroma and their *in vivo* localization is hindered by the lack of known specific surface markers for those cells. To date only a combination of markers can define stem/stromal cells from bone marrow, and none of them is unique. In humans, CD146 (MCAM) has been proposed as a marker for osteogenic cells in stromal tissue capable of establishment of haematopoietic microenvironment, which together meet the definition of mesenchymal stem cell [14]. In mice, a population of CD45^neg^ TER119^neg^ PDGFRa^pos^ Sca1^pos^ sorted cells (PaS) contained multipotent cells that could engraft into MSC compartment after transplantation [15]. However, the clonally derived lines of CD146^pos^ or PaS MSCs exhibited differences in differentiation potentials and transplantability, which may indicate heterogeneity within those cell populations.

Several approaches have been developed for *in situ* localization and lineage tracing of mesenchymal stem cells using mouse transgenic models. It has been shown that in the bone marrow perivascular cells expressing nestin-GFP reporter constitute the haematopoietic niche and contribute to skeletal tissues in lineage-tracing experiments [16]. Recently, Mx1-Cre has been proposed as a marker capable to label the cells that produce osteoblastic lineage life-long in the mice [17]. It was also noted, that Mx1-labelled cells only partially overlap with nestin positive population showing that cellular composition within those pools, each of which display mesenchymal stem cell properties, can be still complex.

Expression of surface antigens in cultured human bone marrow MSCs has been intensively studied by staining with existing antibodies [18], [19], [20], using proteomic approaches [21], [22], [23], [24] and by analysis of their transcriptome [25], [26]. In these works it has been noted that the levels of expression of some surface markers correlates with differentiation capacity of MSC. For example, CD106 and MSCA-1 mainly mark the adipogenic progenitors in cultured human MSCs, whereas ITGA11 is expressed on the cells with osteogenic capacity [20], [26], [27].

Mouse bone marrow MSCs are particularly difficult to characterize due to the low proliferative activity of the cells *in vitro* and substantial contamination of cultures by the cells of haematopoietic origin [28], [29]. At the same time, experience from other stem/progenitor cell types, such as haematopoietic or embryonic, shows that expression of surface epitopes in mouse cells does not always mirror their human counterpart [30], [31]. Since mouse model provides an important platform to study fundamental properties of bone marrow MSCs and is often used for preclinical studies, we were encouraged to perform an extensive study of surface markers in murine MSCs. Previously our group generated a transgenic mouse with inducible expression of SV40 Large T-antigen for conditional immortalization of somatic cells including bone marrow MSCs [32]. Importantly those cells did not change the immunophenotype after long-term culturing, which makes them useful for studies of surface markers. In this report, we characterized the expression of surface markers in conditionally immortalized bone marrow MSC lines using a large panel of antibodies. As a step further we characterized clonally derived MSC progenitors with distinct differentiation potential aiming to find specific markers for MSC subsets, which we could confirm in cell sorting experiments.

## Materials and Methods

### Cell Isolation and Culture

Bone marrow MSCs were derived from transgenic mice with a modified tetracycline-inducible SV40 Large T-antigen generated previously by our group [32]. All protocols related to animal experiments were performed accordingly to the German Animal Welfare Legislation, and the Animal Welfare Officer(s) appointed for the facility oversaw them. For cell isolation bone marrow was flushed from tibia and femurs and plated to cell culture dishes in growth medium DMEM containing 1 g/L D-glucose (Gibco, Life Technologies) supplemented with 10% fetal calf serum (FCS, PAA). After reaching confluency the cells were passaged by diluting 1∶2 without or with adding 10^−7^ M Dexamethasone (Dex) and 1 µg/ml Doxycycline (Dox, both from Sigma) to induce the expression of T-antigen. Conditionally immortalized BM MSCs were routinely cultured in the growth medium in the presence of Dex/Dox and passaged every 3–5 days with dilution 1∶5–1∶10. Cellular cloning was performed by manual dilutions by plating the cells at 1 or 3 cells per well of 96-well plate. After 2 weeks the plates were checked for clones rising from single cells, which were further collected and passaged in 96-well plates using multi-channel pipette. The clones were screened for their potential to differentiate into osteogenic and adipogenic lineages in 96-well plates, at least in triplicates. The clones with distinct differentiation potentials were selected and further expanded.

### Differentiation of BM MSCs

For differentiation assays, BM MSCs were deinduced by Dex/Dox withdrawal for 3 days to stop cell proliferation, and then differentiation conditions were applied to the cells. Osteogenic differentiation was performed in the growth medium by adding 10^−8^ M Dex, 300 µM ascorbic acid and 10 mM β-glycerophosphate. After 2 weeks the cells were fixed with ice-cold methanol for 10 min, stained with 2% Alizarin Red at pH 4.3 for 10 min and dried. For quantification of the efficiency of differentiation the dye was extracted from the stained samples using 4 M guanidine chloride solution at 37°C overnight, and the optical density of extract was measured at 490 nm. For adipogenic differentiation 10^−7^ M Dex, 5 µg/ml insulin and 5 µg/ml Troglitazone were added to the growth medium for 7 days. The cells were fixed with 4% formaldehyde in phosphate buffered saline (PBS) for 30 min at room temperature and stained with Oil Red (0.3% solution in isopropanol mixed 3∶2 with water and filtered) for 5 min. To estimate the efficiency of differentiation the rate of adipocytes was counted using phase contrast microscope at least in three fields of view. Chondrogenic differentiation was done in pellet culture prepared from 10^6^ cells in the medium containing DMEM with 4.5 g/L D-glucose, 1% FCS, 10 ng/ml TGF-β1 (Peprotech), 10^−7^ M Dex, 6.25 µg/ml apo-transferrin, 6.25 µg/ml insulin and 50 µg/ml ascorbate-2-phosphate. After 3 weeks the pellets were fixed in 4% formaldehyde in PBS for 10 min at room temperature, then paraffin embedded and sectioned. The sections were re-hydrated and stained with anti-aggrecan antibody (1∶100, Chemicon) for 1 hour at 37°C. The staining was visualized using Vectastain ABC kit (Vector Labs) according to manufacturer recommendations. All chemicals were purchased from Sigma, unless otherwise stated.

### Cell Surface Marker Screening

BM MSCs were characterized using BD Lyoplate Mouse Cell Surface Marker Screening Panel (BD Biosciences, cat. 562208), which contains 176 monoclonal antibodies and correspondent isotype controls. The staining was done according to manufacturer protocol with minor modifications. Conditionally immortalized MSCs were expanded, and then Dex and Dox were omitted from the growth medium for 3 days to deinduce T-antigen expression. The cells were dissociated using PBS with 10 mM EDTA, washed, filtered through 40 µm nylon mesh and distributed into U-bottom 96-well plates in the concentration 2.5–5×10^5^ cells per well in PBS+2%FCS. The incubation with primary antibodies was done in 50 µl (0.5 µg antibodies per 10^6^ cells) for 30 min at +4°C, followed by two times washing. After that the cells were incubated with the biotinylated secondary antibodies (goat anti-rat 1∶400, goat anti-armenian hamster 1∶800, goat anti-syrian hamster 1∶400 or goat anti-mouse 1∶400) for 30 min at +4°C. After the wash, incubation with Alexa647-Streptavidin conjugate (1∶4000) was done for 30 min at +4°C. The cells were fixed with 2% formaldehyde solution in PBS. The measurement was done using flow cytometer BD LSRII with High-Troughput Screening 96-well plate loader (HTS FACS, BD).

### FACS Sorting

A similar staining protocol was applied for other flow cytometry measurements and cell sorting; the antibodies used for this work were rat anti-mouse CD200 (BD Pharmingen, 552512), rat anti-mouse CD140a (BD Pharmingen, 558774) and mouse anti-mouse SSEA4 (BD Pharmingen, 560073). FACS sorting was done using BD FACS AriaII (BD Biosciences). The data was analyzed using BD FACS Diva and FlowJo (TreeStar) software.

### qRT-PCR

Total RNA was isolated by phenol-chloroform extraction from cell lysate prepared with TRI Reagent (Sigma). 1 µg of RNA was used for DNase I treatment (Invitrogen) and cDNA synthesis (Affinity Script Multiple Temperature cDNA Synthesis kit, Agilent). Quantitative PCR was done with GoTaq qPCR Master Mix (Promega) in 384-well plates using LightCycler480 (Roche). The primers were following (5′-3′): Rpl19 FWD CTGATCAAGGATGGGCTGAT, Rpl19 REV GGCAGTACCCTTCCTCTTCC, PPARg1 FWD TGAAAGAAGCGGTGAACCACTG, PPARg1 REV TGGCATCTCTGTGTCAACCATG, CD200 FWD CTGGAAACGTCACCGAAATC, CD200 REV TCCCTCCTGCTTTTCTTTCA, Sp7 FWD TCCTCGGTTCTCTCCATCTG, Sp7 REV GGACTGGAGCCATAGTGAGC, Alpl FWD GGGACGAATCTCAGGGTACA, Alpl REV TTCAAGGTCTCTTGGGCTTG, Plin4 FWD GCCACATACAGCACAACCAG, Plin4 REV CTACCAACAGCCTCCACCAT, CD140a FWD ACAGAGACTGAGCGCTGACA, CD140a REV CGATGGTCTCGTCCTCTCTC, AP2 FWD GATGGTGACAAGCTGGTGGT, AP2 REV AATTTCCATCCAGGCCTCTT.

### Western Blot

Whole cell protein extracts were prepared by freezing and thawing of cells in the extraction buffer (20 mM Hepes Ph 8.0, 350 mM NaCl, 10% glycerol, 0.1% Tween-20, 2 mM EDTA, 1 mM DTT, 1 mM PMSF, 1% Protease Inhibitor Coctail, all Sigma). 20 µg of proteins were resolved in PAGE gel and transferred to nitrocellulose membrane using semi-dry blotting. Blocking was done overnight in 5% milk in PBS with 0.1% Tween-20 at +4°C. The membranes were incubated with primary antibodies (mouse monoclonal against T-antigen, 1∶1000, Santa Cruz) for 1 hour at room temperature, washed and incubated with secondary horseradish peroxidase-conjugated goat anti-mouse antibodies (1∶2000, Pierce Thermo Scientific) in the same conditions. Detection was performed using SuperSignal West Femto Substrate kit (Pierce Thermo Scientific).

## Results

### Characterization of Conditionally Immortalized Mouse Bone Marrow MSCs

**Figure 1:**
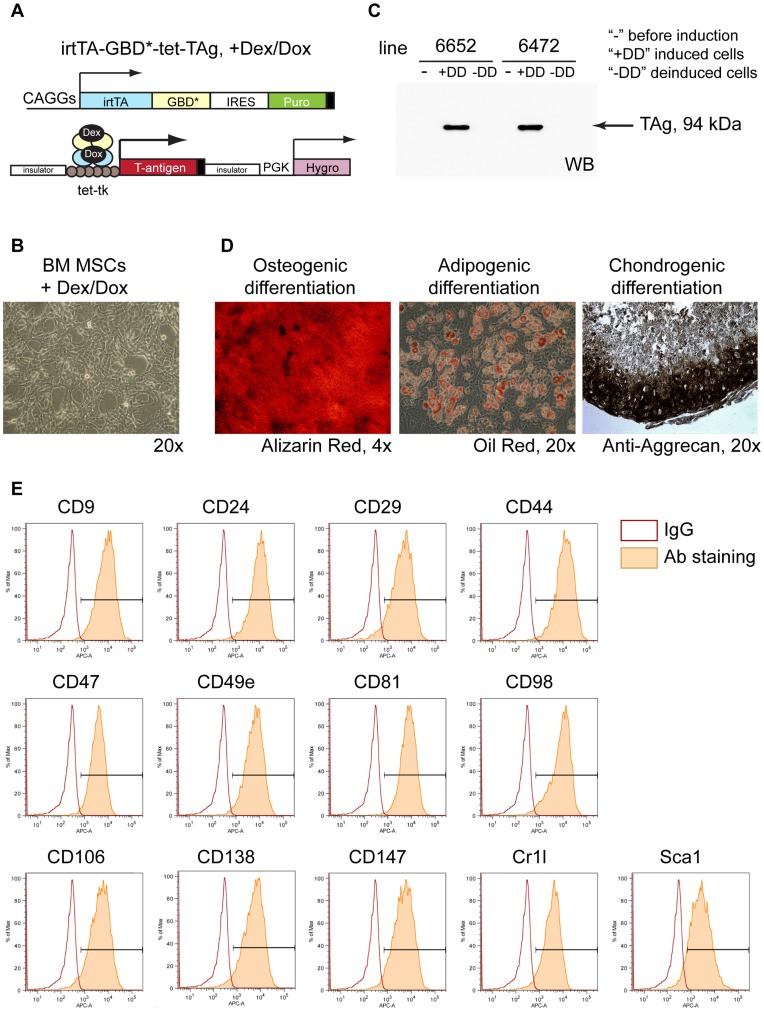
Characterization of conditionally immortalized mouse bone marrow MSCs. (A) An improved tet-inducible system for conditional expression of Large T-antigen. In the presence of Dex/Dox irtTA-GBD* fusion protein can be translocated to the nucleus and activate transcription of Large T. (B) MSCs were isolated from bone marrow of transgenic mice and expanded in the immortalization conditions (Phase Contrast, 20×). (C) Western blot showing induction of T-antigen in primary MSCs (“−“ cells before induction, “+DD” induced cells, “−DD” cells deinduced for 3 days). (D) Conditionally immortalized MSCs maintained differentiation potential *in vitro* into osteo-, adipo- and chondrogenic lineages. (E) Flow cytometry results for the markers highly expressed in MSCs, as shown by the staining with BD Lyoplate Antibody Screening Panel. Abbreviations: Dex – Dexamethasone, Dox – Doxycycline, irtTA – improved reverse tetracycline transactivator, GBD* - mutated glucocorticoid-binding domain, TAg – Large T-antigen, CAGGs and PGK – constitutively active promoters, tet-tk – tetracycline operator and minimal promoter, IRES – internal ribosome binding site, Puro and Hygro – puromycin and hygromycin resistance genes.

To establish expandable MSCs we isolated cells from the bone marrow of transgenic mice carrying a modified system for tetracycline-regulated expression of SV40 Large T-antigen, which has been reported previously by our group [32], [33]. The expression of Large T was induced in primary MSCs in the presence of two ligands, Dexamethasone and Doxycycline (Dex and Dox), resulting in rapid cell proliferation (Fig. 1A, B). The conditionally immortalized cells could be passaged practically indefinitely, whereas uninduced cells had limited growth potential and exhibited signs of senescence after 1–3 passages [32]. After removing Dex/Dox from the medium, T-antigen was not expressed anymore (Fig. 1C), and consequently the cells stopped dividing, showing that immortalization was reversible. Deinduced cells could be differentiated into osteogenic, adipogenic and chondrogenic lineages (Fig. 1D) by applying culture conditions described for MSCs, indicating that immortalization did not affect the differentiation potential of MSCs.

To characterize the expression of surface markers in mouse bone marrow MSCs we used conditionally immortalized lines from two individual mice (#6472 and #6652) for screening using an antibody panel BD Lyoplate and high-throughput flow cytometry. The immortalization was deinduced by Dex/Dox withdrawal for 3 days before the experiment to avoid influence of T-antigen expression to the cell properties. The Mouse Cell Surface Marker Screening Panel contains 176 specific monoclonal antibodies and corresponding isotype controls. Amongst the markers present in the panel we identified 13 antigens that were homogeneously and highly expressed on the surface of bone marrow MSCs, with more than 85% of cells in the positive gate (Table 1, Fig. 1E). These included CD9 (Tetraspanin 29), CD24 (Heat Stable Antigen), CD29 (Integrin beta-1), CD44 (Hyaluronic acid receptor), CD47 (Integrin-associated protein), CD49e (Integrin alpha-5), CD81 (Tetraspanin 28), CD98 (Slc3a2), CD106 (Vcam1), CD138 (Syndecan-1), CD147 (Basigin), Cr1l (complement component 3b/4b receptor 1-like), Sca1 (Stem Cell Antigen 1).

**Table 1:** Immunophenotype of conditionally immortalized mouse BM MSCs.

Marker	Alternate name	% of positive	Ref
*Highly expressed*			
CD9	Tspan29, Tetraspanin 29	99.1	[25]
CD24	HSA, Heat Stable Antigen	98.6	[18] *
CD29	Itgb1, Integrin beta-1	90.7	[25], [18], [50] *
CD44	Hyaluronate receptor	99.0	[25], [51], [18], [50] *
CD47	Itgp, Integrin-associated protein	97.6	[21], [25]
CD49e	Itga5, Integrin alpha-5, Fibronectin receptor	97.5	[25], [51], [18] *
CD81	Tspan28, Tetraspanin 28	97.8	[25], [51], [18] *
CD98	Slc3a2, Solute Carrier family 3 member 2	95.3	[21]
CD106	Vcam1, Vascular Cell Adhesion Molecule 1	92.1	[25], [28] *
CD138	Sdc1, Syndecan-1	94.8	**N**
CD147	Bsg, Basigin	94.0	[51]
Cr1l	Complement component (3b/4b) receptor 1-like, Crry, p65	93.8	**N**
Sca1	Ly6a, Stem Cell Antigen-1	85.3	[18], [50], [52] *, +/− [28] *
*Moderately or heterogeneously expressed*			
CD13	Anpep, Aminopeptidase N	6.4	[25]
CD51	Itgav, Integrin alpha-v, Vitronectin receptor	52.8	[21], [25], [18] *
CD61	Itgb3, Integrin beta-3	30.7	[25], [18] *
CD71	Tfrc, Transferrin receptor	55.1	[21], [18] *
CD73	Nt5e, Ecto-5′-nucleotidase	28.1	[25]
CD80	T-lymphocyte activation antigen CD80	20.1	[18] *
CD95	Fas, TNF receptor superfamily member 6	11.8	[18] *
CD107b	Lamp2, Lysosomal-Associated Membrane Protein 2	9.7	[21]
CD119	Ifngr1, Interferon Gamma Receptor Alpha	60.1	[53]
CD120a	Tnfrsf1a, TNF Receptor superfamily member 1a	11.2	[54]
CD120b	Tnfrsf1b, TNF Receptor superfamily member 1b	38.9	[25], [18] *
CD140a	Pdgfra, PDGF Receptor alpha	14.5	[25], [51], [18], [52] *
CD172a	Sirpa, Signal-Regulatory Protein alpha	7.8	[55]
CD200	OX-2	16.0	[25]
IFNgR1b	Interferon gamma receptor 1, beta chain	8.5	[53]
Sdc4	Syndecan-4	58.5	**N**
Ly-51	Enpep, Glutamyl Aminopeptidase, CD249	34.6	[56]
H-2K^b^	H-2Kb MHC class I alloantigen	60.5	[50] *
H-2D^b^	H-2Db MHC class I alloantigen	67.0	**N**
SSEA-4	Stage Specific Embryonic Antigen 4	11.2	[25], [46] *

* reported for mouse BM MSCs.

+/−reported as inconsistent level of expression between MSC preparations.

**N**-not reported for expression in MSCs before.

We detected 20 markers, which were moderately expressed or showed heterogeneous staining within MSC population with more than 5% of cells in the positive gate (Table 1, Fig. S1).

All the other antibodies used for the screening did not show a positive staining (143 markers). Notably, we did not detect expression of haematopoietic markers, such as CD11b, CD19, CD34, CD45, corresponding to characteristics of MSCs and confirming the absence of contamination with the cells of blood lineages. The immunophenotype of bone marrow MSCs was essentially reproduced in two lines that we used for the screening. The complete results of the screening are shown in (Fig. S1, S2).

### Characterization of Bone Marrow MSC Subpopulations

**Figure 2:**
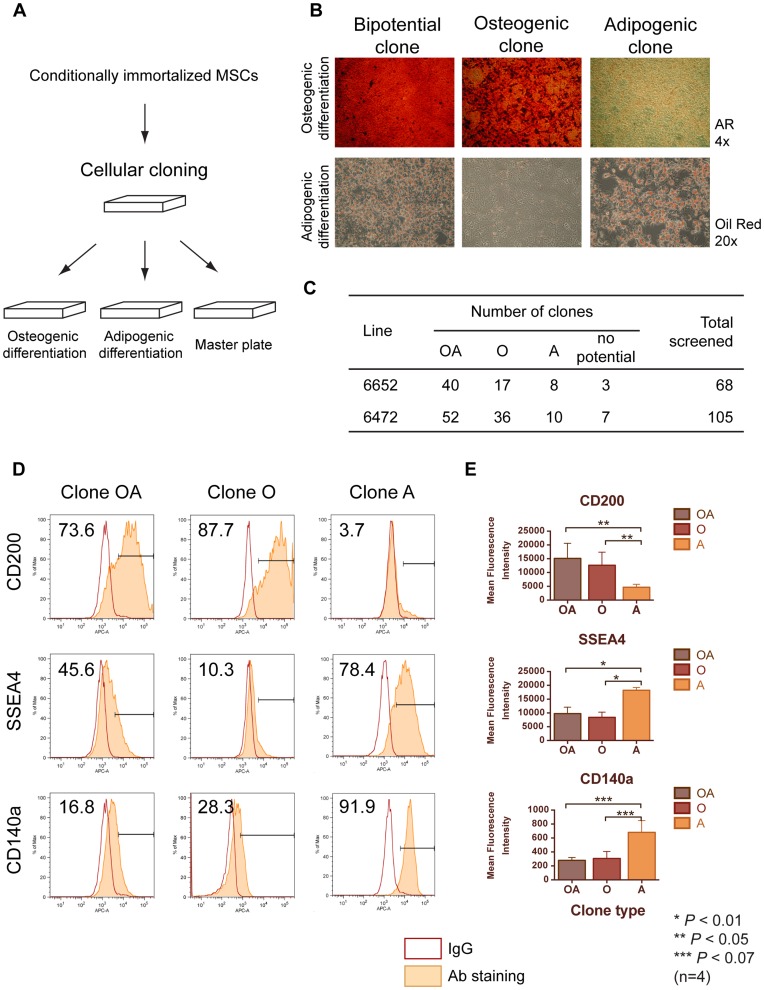
Characterization of clonally derived bone marrow MSC progenitors. (A) Cellular cloning of conditionally immortalized MSCs was performed by limiting dilutions, and the resulted single-cell derived subpopulations were cultured in 96-well plates and screened for the potential for osteogenic and adipogenic differentiation. (B) Differentiation assay revealed the bipotential clones capable of osteo- and adipogenesis “OA”, and monopotential osteo- and adipogenic clones, “O” and “A”. (C) Summary of screening of two independent MSC lines. (D) Flow cytometry results of staining of clonally derived MSC progenitors with OA, O and A potential, for the differentially expressed markers CD200, SSEA4 and CD140a. The percent of cells in the positive gate is indicated on the histogram. (E) Comparison of levels of differentially expressed markers CD200, SSEA4 and CD140a shown by Mean Fluorescence Intensity of flow cytometry measurement, between OA, O and A progenitors. 4 clones of each type were used for experiment, statistical significance is indicated.

We aimed to dissect a population of MSCs at the single-cell level and characterize progenitors committed to different lineages focusing on the osteogenic and adipogenic properties. For this purpose we performed cellular cloning of conditionally immortalized MSCs and established clonally derived subpopulations from MSC lines derived from two individual mice (#6472 and #6652). The clones were screened for their potential to differentiate into osteocytes and adipocytes (Fig. 2A). The differentiation assays were done in at least 3 independent experiments and only completely reproducible results were considered for the analysis. We identified bipotential clones capable for both types of differentiation (OA), as well as monopotential progenitors for osteo- and adipogenic lineages, O and A, respectively (Fig. 2B, C).

To characterize the expression of surface markers in different types of MSC progenitors we performed a screening by staining with Lyoplate Antibody Panel and flow cytometry, considering only those antigens that we found to be positive or moderately positive/heterogeneous in the initial MSC lines (33 antibodies). For each MSC subsets (OA, O, A) we chose 4 representative clones, two originating from each of two mice (in total 12 clones).

All clones were positive for the markers, which were highly and uniformly expressed in the original MSC lines, listed in Table 1. Of a particular interest was to analyze the expression of markers that showed heterogeneous staining in the parental lines. Most of those antigens exhibited similar intensity of staining to the MSC lines and amongst the clones. But we also detected differences in the expression of three antigens between MSC subsets, and among them CD200 (OX-2), SSEA4 (stage-specific embryonic antigen 4) and CD140a (platelet-derived growth factor receptor alpha), (Fig. 2D, E). CD200 was expressed higher in all clones with osteogenic potential (OA and O) than in the A monopotential clones, 3.3- and 2.7-fold (*P*<0.05), respectively, by comparing the Mean Fluorescence Intensity (MFI). Conversely, SSEA4 was represented more in the A clones with MFI of 1.9- and 2.2-fold higher relatively to OA and O clones (*P*<0.01). A similar pattern was found for CD140a, which was 2.4- and 2.2-fold higher expressed in the A clones compared to OA and O, respectively, although with lower significance (*P*<0.07).

It has to be noted that we detected the presence of all three antigens in most of the analyzed clones, but the levels of expression correlated with the differentiation properties of the cells (Fig. 2D). Therefore, we proposed CD200, SSEA4 and CD140a as differentially expressed between bone marrow MSC progenitors committed to different lineages.

### Comparison of Bone Marrow MSC Subsets Sorted for CD200, SSEA4 and CD140a Expression

**Figure 3:**
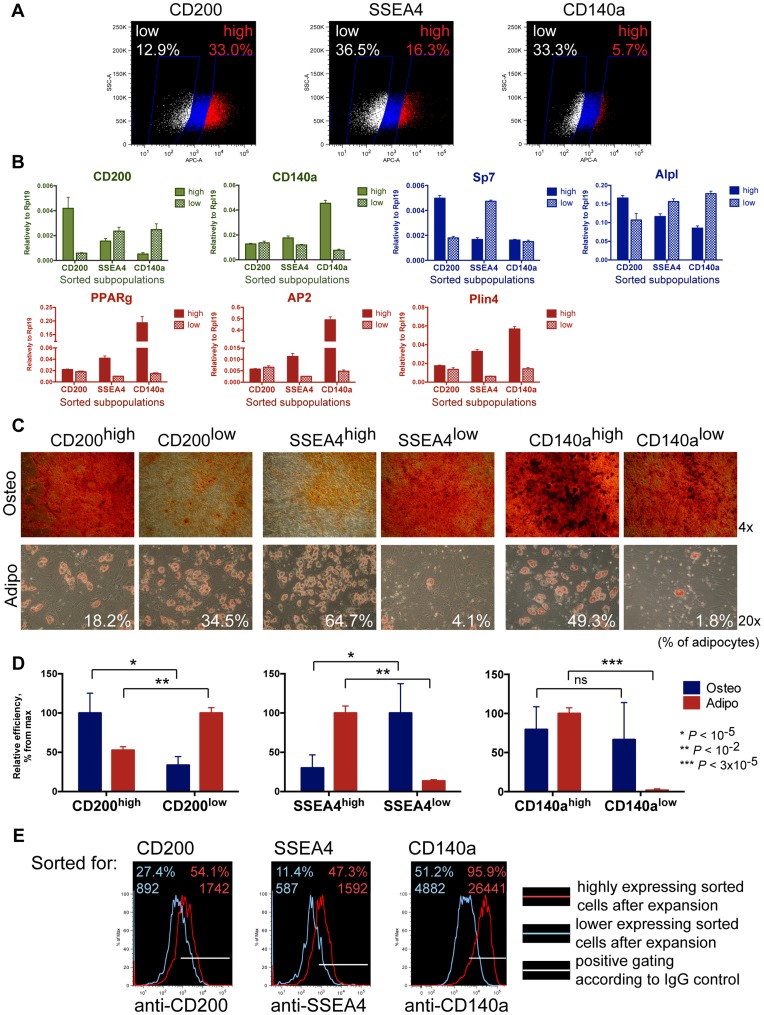
Cellular sorting of bone marrow MSC subsets with different levels of CD200, SSEA4 and CD140a expression. (A) FACS gates for sorting of MSC subpopulations for high and low expression of CD200, SSEA4 and CD140a. (B) Quantitative RT-PCR for expression of markers used for sorting, CD200 and CD140a (green color), and lineage-specific markers (osteogenic in blue and adipogenic in red), in the cells directly after sorting. (C) The sorted subpopulations were differentiated into osteocytes (stained with Alizarin Red, 4×) and adipocytes (stained with Oil Red, 20×, percent of adipocytes is indicated). (D) Comparison of osteogenic and adipogenic potentials in the sorted MSC subpopulations. Efficiency of osteogenic differentiation was quantified by Alizarin Red extraction and measurement of OD at 490 nm of the eluate, and adipogenic differentiation was assessed by adipocyte counting. High- and low-expressing markers subsets were compared and the percent of differentiation efficiency from the maximal among them is shown. (E) Flow cytometry results of MSC subpopulations for the markers, which were used for sorting. After at least 5 passages in culture the positively sorted cells (red line) maintained elevated level of expression compared to the negatively sorted cells (blue line).

To validate differential expression of CD200, SSEA4 and CD140a in distinct types of bone marrow MSC progenitors we decided to separate MSC subsets with high and low levels of those markers and test the resulted subpopulations for differentiation potential. We stained two conditionally immortalized mouse bone marrow MSC lines (#6472 and #6652) individually with the antibodies for CD200, SSEA4 and CD140a, and sorted distinct subpopulations with high and low expression of those markers by FACS (Fig. 3A). The obtained subpopulations were checked for the level of CD200 and PDGFRa mRNAs by qRT-PCR immediately after sorting (Fig. 3B), SSEA4 has no correspondent transcript, as it is an oligosaccharide. As expected, CD200^high^ sorted population expressed 7.1-fold higher level of CD200 as mRNA, and CD140a^high^ cells had 5.9-fold more of PDGFRa, confirming the efficiency of the sorting. We also noted that SSEA4^high^ cells expressed 1.5 times higher CD140a and 1.5 times lower CD200 than SSEA4^low^ subset. Additionally, we tested the levels of lineage-specific markers in the cells, characteristic for osteogenic (Sp7 and Alpl) and adipogenic commitment (PPARg, AP2, Plin4). We confirmed higher expression of osteogenic markers by CD200^high^ compared to CD200^low^ subpopulations (2.8-fold for Sp7 and 1.5-fold for Alpl), whereas PPARg, AP2 and Plin4 were present at similar levels. On the opposite, the SSEA4^high^ MSC subpopulation had upregulated adipogenic marks (4.2- to 5.5-fold) and downregulated osteogenic (2.8-fold for Sp7 and 1.3-fold for Alpl) relatively to SSEA4^low^. CD140a^high^ cells expressed remarkably higher levels of adipogenic markers than CD140a^low^ (13.4-fold for PPARg, 102-fold for AP2 and 4-fold for Plin4), whereas osteogenic markers Sp7 and Alpl were not consistently changed.

The sorted subpopulations were expanded in the immortalization conditions and further tested for their differentiation capacity (Fig. 3C, D). We found that CD200^high^ MSCs had higher osteogenic (*P*<10^−5^) and lower adipogenic potential (*P*<10^−2^) compared to CD200^low^ subset. The cells sorted for high SSEA4 expression exhibited more efficient adipogenic (*P*<10^−5^) and less efficient osteogenic differentiation (*P*<10^−2^) than SSEA4^low^ cells. CD140a^high^ subpopulation was more adipogenic relatively to CD140a^low^ (*P*<3×10^−5^), whereas osteogenic properties varied between subsets isolated from different mice and did not show consistent difference. Generally, the differentiation capacity of the expanded sorted subpopulations was in concordance to the expression of lineage-specific markers on mRNA level, defined by qPCR immediately after sorting. These results were reproduced in the experiments using two independent mouse MSC lines.

We compared the levels of all three markers CD200, SSEA4 and CD140a by FACS in the high/low sorted subpopulations after expansion for at least 5 passages after sorting (Fig. 3E, Fig. S3). CD200^high^ cells expressed 2.2-fold higher level of CD200 (*P*<0.01) and 1.4-fold lower of SSEA4 (*P*<0.02) compared to CD200^low^ population. Conversely, SSEA4^high^ cells showed 1.9-fold higher expression of SSEA4 and 1.6-fold lower of CD200 relatively to SSEA4^low^. The level of CD140a was not significantly different between CD200^high^/CD200^low^ and SSEA4^high^/SSEA4^low^ populations. Corresponding to that, CD140a^high^ cells did not exhibit major differences in expression of CD200 and SSEA4 markers to CD140a^low^, and at the same time had 4.5-fold upregulated CD140a.

Summarizing those results, a high level of CD200 marks MSC progenitors with elevated osteogenic potential, whereas SSEA4 is expressed in the adipogenic cells, and high expression of those markers is mutually exclusive. These results were confirmed by the staining with combination of these antibodies (Fig. S4). CD140a is present at high level in adipogenic cells, but its expression does not exclude the osteogenic properties in this subpopulation.

### Expression of Markers during Differentiation of Bone Marrow MSCs

In order to check whether the markers differentially expressed in the MSC progenitors with distinct lineage commitment, are associated with differentiation process, we tested expression of CD200, SSEA4 and CD140a during the time course of osteogenic and adipogenic induction.

**Figure 4:**
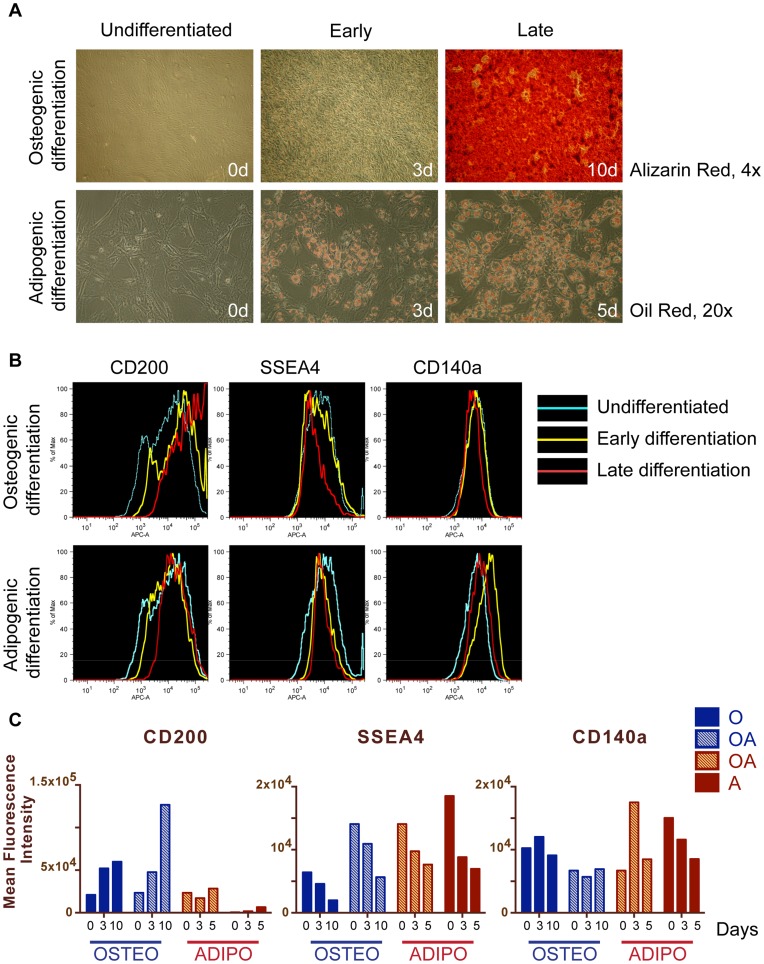
Expression of CD200, SSEA4 and CD140a during MSC differentiation. (A) Clonally derived conditionally immortalized MSCs were differentiated into osteocytes (upper panel, Alizarin Red staining) or adipocytes (lower panel, Oil Red staining). Undifferentiated state, early time point and late differentiation were analyzed (0, 3, 10 days of osteogenic induction, and 0, 3, 5 days of adipogenic induction). (B) Flow cytometry results of the expression of CD200, SSEA4 and CD140a in the representative OA clone during differentiation time course (0, 3, 10 days of osteogenic treatment and 0, 3, 5 days of adipogenic treatment). (C) Dynamics of markers expression in the MSC progenitors during osteogenic differentiation (O and OA clones, blue and blue patterned bars, respectively), and adipogenic differentiation (the same OA and A clone, red patterned and red bars), shown as Mean Fluorescence Intensity from flow cytometry measurements. CD200 was upregulated during osteogenic induction, while SSEA4 was downregulated in all differentiation conditions. CD140a reached a high level during adipogenesis in the OA clone, and then dropped, and in the A clone CD140a downregulated from high level during differentiation.

We performed osteogenic differentiation with two clones of the OA type and two O clones, and adipogenic differentiation with the same two OA and two A clones (Fig. 4A). The expression of markers was checked in undifferentiated cells (day 0), during the time course of differentiation (days 3 and 5 for adipogenesis, days 3, 5 and 10 for osteogenesis, for the latter only days 3 and 10 are shown), (Fig. 4B, C). Flow cytometry results showed that CD200 was upregulated during osteogenic induction of OA and O clones, but stayed on the similar level during adipogenesis. SSEA4 was gradually downregulated in all clones in differentiation conditions. CD140a did not change during osteogenic differentiation. During adipogenic treatment of the OA clones CD140a peaked on the day 3 and then dropped, but it was downregulated from early stages of adipogenic induction of the A clones, which had had a high level of CD140a before applying differentiation conditions.

## Discussion

Functional studies of specific cell types are inevitably based on the ability to visualize the cells in the tissue context or to isolate pure cell populations and estimate their homogeneity. Isolation of HSCs expressing the set of well-established surface antigens [34], [35] and localizing intestinal stem cells using Lgr5 reporter [36] provided extraordinary examples of breaking through in these fields of research. Unfortunately, those stem cell types are exceptions among many others existing in mammalian body. Despite accumulating knowledge on the location and functions of bone marrow mesenchymal stem cells [14], [16], [17], a concise view on their identity is still missing and furthermore the hierarchy of their descendants is not defined.

*In vitro* culture systems represent a model to study cell properties and can provide sufficient cell numbers for biochemical characterization, although caution should be taken because of possibly introduced artifacts. It has been previously noted that cultured human BM MSC are changing their differentiation properties and expression of markers during passaging [27], [37], [38], which hampers the studies of their properties and makes characterization of BM MSC subtypes complicated. Moreover, mouse BM MSCs are difficult to expand [28], [39] and therefore are not well described. We applied conditional immortalization for BM MSC expansion by isolating the cells from transgenic mice carrying inducible SV40 Large T-antigen [32], [33]. This system enables expression of Large T in the presence of two compounds, Dex and Dox, which is reversible after withdrawal of ligands. In our previous work we have shown that immunophenotype of conditionally immortalized MSCs is not changed after prolonged culturing and upon induction/deinduction [32]. These observations motivated us to perform an extensive characterization of the expression of surface antigens in conditionally immortalized BM MSCs. Here we report the results on BM MSC immunophenotyping using 176 antibodies, and to our knowledge this is the most comprehensive study of surface markers expressed in mouse bone marrow MSCs to date (Fig. 1, Table 1, Fig. S1, S2). Among 33 antigens that we detected in conditionally immortalized MSCs, 13 markers have not been previously characterized in mouse BM MSCs. Additionally, 3 markers (CD138, Cr1l, Sdc4) have not been reported for BM MSCs neither from mice nor from other species.

Conditionally immortalized BM MSCs have practically indefinite proliferative potential, which allowed us establishing clonally derived subpopulations. We found progenitors with distinct differentiation potential among BM MSC subpopulations (Fig. 2), which confirms heterogeneity of MSCs reported by others [38], [40], [41], [42]. By comparing immunophenotypes of clonally derived progenitors we showed that osteogenic cells (with OA and O properties) expressed higher level of CD200 (OX2) and lower levels of SSEA4 and CD140a (PDGFRa) than non-osteogenic (A clones). Our sorting experiments confirmed that expression of those markers is distinctive for subpopulations with different efficiencies of osteogenesis and adipogenesis (Fig. 3).

We showed that high expression of CD200 marks the progenitors with higher potential for osteogenesis in expense of adipogenic capacity. Our observations are in a good agreement with the molecular function of CD200, which is to mediate osteoblast-osteoclast interaction and to take part in regulation of balance between bone formation and resorption [43], [44]. Lee et al. [43] reported that CD200 enhanced differentiation of primary calvarial osteoblasts upon overexpression or after adding a soluble form of CD200 to the cells. Our experiments showed that CD200 was upregulated during osteogenesis in BM MSCs, which may indicate positive autoregulation during differentiation. Another study suggested CD200 as a marker for multipotent human BM MSCs characteristic for undifferentiated cells [25]. The authors described a reduction in CD200 level after osteogenic and adipogenic differentiation of BM MSCs, which contradicts to our data and observations of the others [43]. We cannot explain this discrepancy, however the difference in the role of CD200 in human and mouse MSCs cannot be completely excluded.

We found that SSEA4 was characteristic for the cells with higher adipogenic potential and lower osteogenic potential, which was the opposite distribution to CD200 within BM MSC populaiton. Detection of SSEA4 in our study is remarkable since this molecule represents a globo-series glycosphingolipid and would not be detected by transcriptomic or proteomic methods [45]. Expression of SSEA4 in human and mouse BM MSCs was previously described [46], and it was shown as heterogeneous in expanded primary BM MSCs. SSEA4 positive cells were found to be tripotential (osteo-, adipo-, chondrogenic), although with a low mineralization efficiency, but unfortunately, a side by side comparison with SSEA4 low expressing cells hasn’t been done.

We identified CD140a to be higher expressed in adipogenic monopotential progenitors than in osteogenic, and further confirmed higher adipogenic efficiency of CD140a^high^ subpopulation, but osteogenic property of CD140a^high^ and CD140a^low^ cells was variable. Therefore, CD140a expression level did not correlate with the capacity for the alternative differentiation pathway, in contrast to the other markers analyzed in our work. Indeed, we observed that CD140a was expressed among MSCs independently of SSEA4 level (Fig. S4). CD140a was used for prospective isolation of MSCs from mouse bone marrow [15]. In this work, CD45^neg^TER119^neg^ cells from bone marrow were separated into subpopulations with different expression of PDGFRa and Sca1 and checked for differentiation potential. Interestingly, only PDGFRa^pos^ cells exhibited adipogenic potential (both Sca1^pos^ and Sca1^neg^). This observation supports our data obtained using *in vitro* cultured cells. Additionally, we found that CD140a reached high level on the early stage of adipogenesis, also in the OA clones that had initially a low level of CD140a, and then dropped. Similar to this result, during adipogenic differentiation of mouse embryonic stem (ES) cells PDGFRa expressing cells were giving rise to adipocytes, but not PDGFRa^neg^ [47]. It was also shown that adding PDGF inhibited differentiation of pre-adipocytes 3T3-L1 [48] and blocking of PDGF receptor by antibodies promoted adipogenesis in MSCs [49]. Taking together these data and our results, we suggest that high level of PDGFRa marks adipogenic precursors at the early stage of commitment, but has to be downregulated to proceed to terminally differentiated state.

In conclusion, we performed a comprehensive characterization of surface marker expression in mouse bone marrow MSCs and their subsets using conditionally immortalized cells. Moreover, we were able to identify the markers differentially expressed in the distinct types of BM MSC progenitors, CD200 in osteogenic and SSEA4 and CD140a in adipogenic cells. Our results conform to the data obtained using primary cells and mice [15], [43], [47], and provide an important insight to the identity of BM MSCs and cellular composition of this cell population. We believe that our findings would be effective for the studies of bone marrow stroma functions and understanding biology of mesenchymal progenitor cells: further investigations are anticipated.

## Supporting Information

Figure S1 **Expression of surface markers in conditionally immortalized mouse BM MSCs.** Expression of 176 markers was checked by staining with antibodies and flow cytometry of two MSC lines after de-induction of immortalization. The results for 13 highly and 20 moderately/heterogeneously expressed antigens are shown as histograms and percents of cells in the positive gate are indicated (for one of the lines). Line – IgG control, colored histogram – antibody staining.
(TIF)

Figure S2 **The complete results of surface markers screening in conditionally immortalized mouse BM MSCs.** Three 96-well plates containing antibodies, which were used for the staining, are depicted (the empty wells contained isotype controls or nothing). The color indicates the result of the measurement as shown; the average percentage of cells in the positive gate was calculated from the screening of two individual lines.
(TIF)

Figure S3 **Expression of surface markers in the BM MSC subsets sorted for CD200, SSEA4 and CD140a.** The subpopulations of BM MSCs were sorted from two individual conditionally immortalized lines for high and low expression of the markers above, passaged for at least 5 times and each of them was checked for the levels of all those three markers by flow cytometry. The Whisker box plots show the ratio of the Mean Fluorescence Intensity (MFI) in the population sorted for high level to MFI in the one sorted for low expression of (A) CD200, (B) SSEA4 and (C) CD140a. The dashed line represents ratio = 1, i. e. equal expression. The results are summarized from two independent measurements of subsets sorted from two lines. Statistical significance was calculated using Student’s t-test. The cells sorted for high expression of CD200, SSEA4, CD140a maintained increased level of those markers as compared to the cells sorted for low expression. Additionally, CD200^high^ subpopulation exhibited lower level of SSEA4 compared to CD200^low^, whilst SSEA4^high^ had decreased CD200 expression relatively to SSEA4^low^.
(TIF)

Figure S4 **Co-expression of markers CD200 and SSEA4 or CD140a and SSEA4 in conditionally immortalized BM MSCs.** Conditionally immortalized BM MSCs were stained with combinations of antibodies for CD200 and SSEA4 (upper panel) or CD140a and SSEA4 (lower panel). The gating has been done according to unstained control and stainings with individual antibodies combined with all secondary reagents to exclude unspecific staining. BM MSCs were mostly composed of the subpopulations with CD200^high^ SSEA4^low^ and CD200^low^ SSEA4^high^ immunophenotypes and high expression of both markers was exclusive. Expression of CD140a was detected within SSEA4^high^ and SSEA4^low^ subsets. A percentage and Mean Fluorescence Intensity for PE and APC staining are shown for the described subpopulations.
(TIF)
