# Supplementary figures and images for: Differential Expression of Surface Markers in Mouse Bone Marrow Mesenchymal Stromal Cell Subpopulations with Distinct Lineage Commitment
Source: PLoS One. 2012 Dec 7;7(12):e51221. doi: 10.1371/journal.pone.0051221 (PMC3517475; doi:10.1371/journal.pone.0051221)

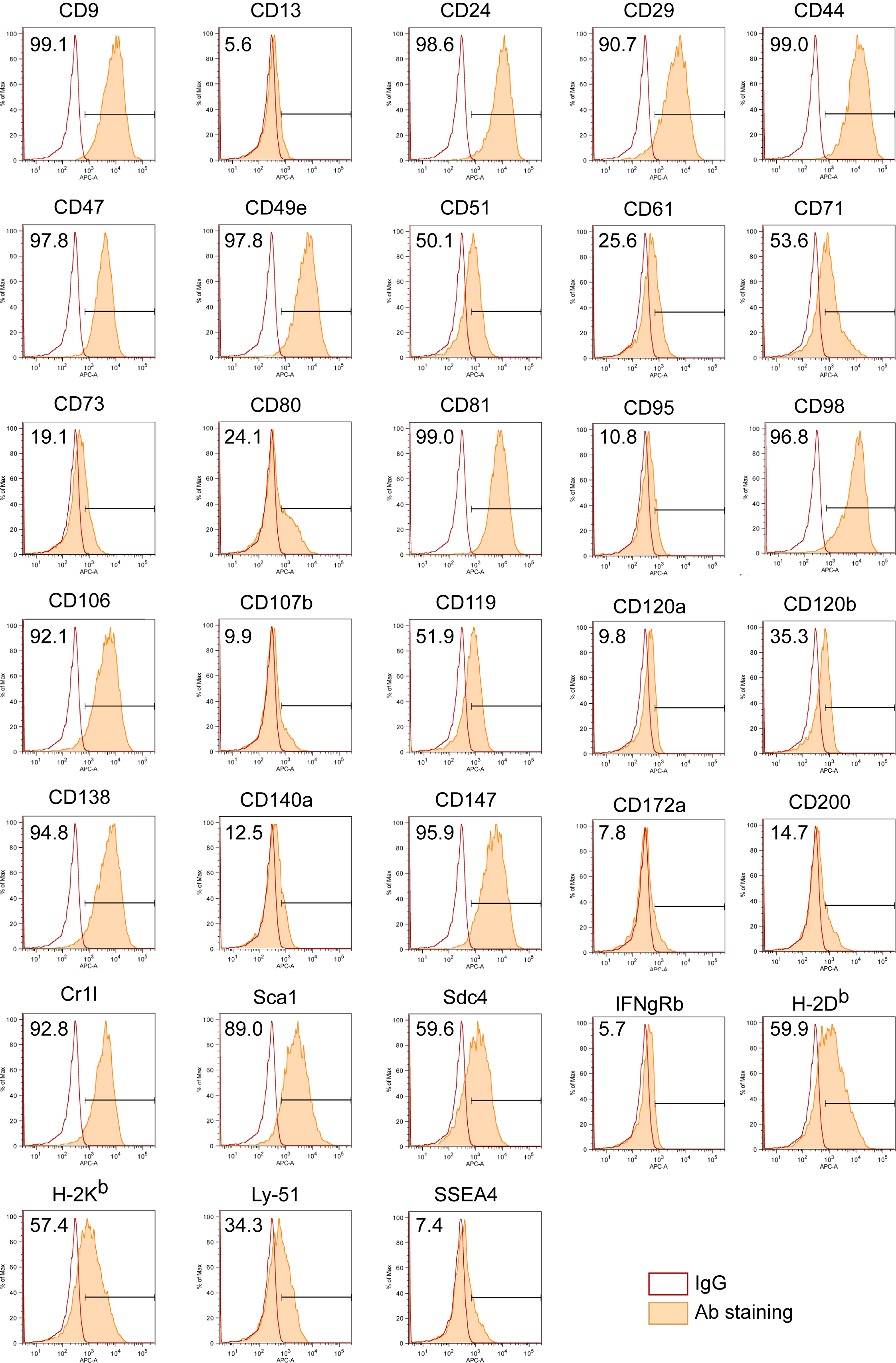

Supplement: Figure S1 — Expression of surface markers in conditionally immortalized mouse BM MSCs. Expression of 176 markers was checked by staining with antibodies and flow cytometry of two MSC lines after de-induction of immortalization. The results for 13 highly and 20 moderately/heterogeneously expressed antigens are shown as histograms and percents of cells in the positive gate are indicated (for one of the lines). Line – IgG control, colored histogram – antibody staining. (TIF) [file pone.0051221.s001.tif]

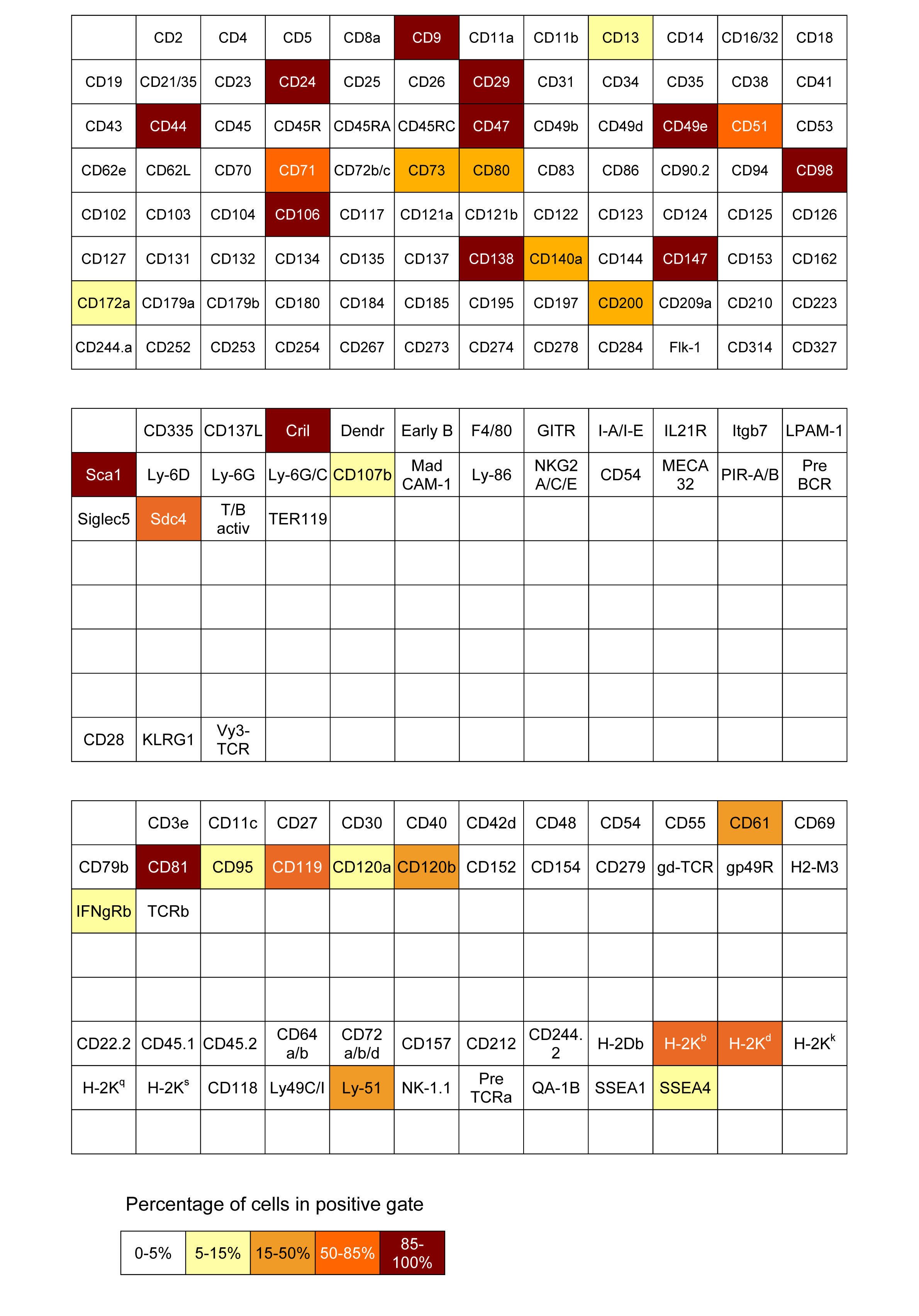

Supplement: Figure S2 — The complete results of surface markers screening in conditionally immortalized mouse BM MSCs. Three 96-well plates containing antibodies, which were used for the staining, are depicted (the empty wells contained isotype controls or nothing). The color indicates the result of the measurement as shown; the average percentage of cells in the positive gate was calculated from the screening of two individual lines. (TIF) [file pone.0051221.s002.tif]

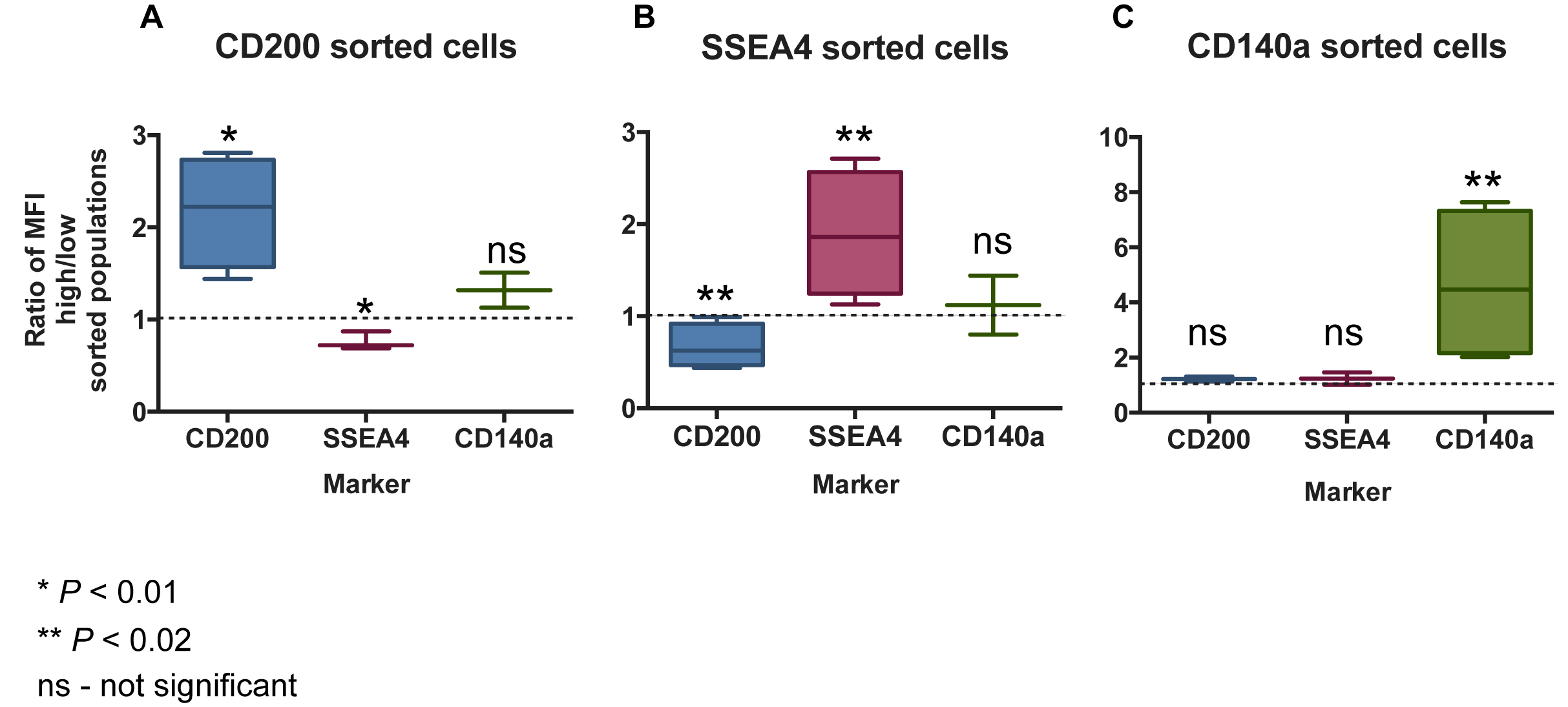

Supplement: Figure S3 — Expression of surface markers in the BM MSC subsets sorted for CD200, SSEA4 and CD140a. The subpopulations of BM MSCs were sorted from two individual conditionally immortalized lines for high and low expression of the markers above, passaged for at least 5 times and each of them was checked for the levels of all those three markers by flow cytometry. The Whisker box plots show the ratio of the Mean Fluorescence Intensity (MFI) in the population sorted for high level to MFI in the one sorted for low expression of (A) CD200, (B) SSEA4 and (C) CD140a. The dashed line represents ratio = 1, i. e. equal expression. The results are summarized from two independent measurements of subsets sorted from two lines. Statistical significance was calculated using Student’s t-test. The cells sorted for high expression of CD200, SSEA4, CD140a maintained increased level of those markers as compared to the cells sorted for low expression. Additionally, CD200high subpopulation exhibited lower level of SSEA4 compared to CD200low, whilst SSEA4high had decreased CD200 expression relatively to SSEA4low. (TIF) [file pone.0051221.s003.tif]

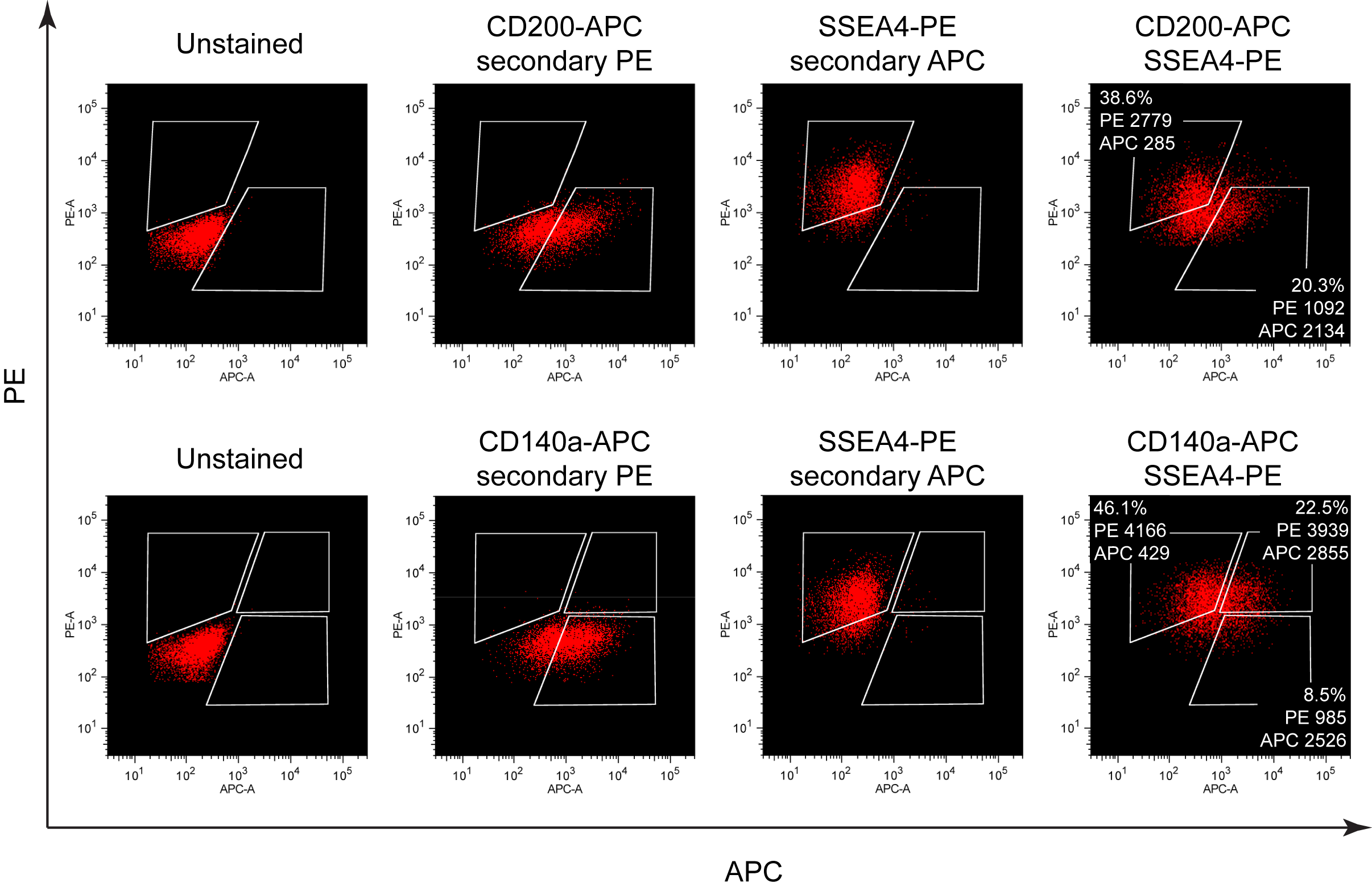

Supplement: Figure S4 — Co-expression of markers CD200 and SSEA4 or CD140a and SSEA4 in conditionally immortalized BM MSCs. Conditionally immortalized BM MSCs were stained with combinations of antibodies for CD200 and SSEA4 (upper panel) or CD140a and SSEA4 (lower panel). The gating has been done according to unstained control and stainings with individual antibodies combined with all secondary reagents to exclude unspecific staining. BM MSCs were mostly composed of the subpopulations with CD200high SSEA4low and CD200low SSEA4high immunophenotypes and high expression of both markers was exclusive. Expression of CD140a was detected within SSEA4high and SSEA4low subsets. A percentage and Mean Fluorescence Intensity for PE and APC staining are shown for the described subpopulations. (TIF) [file pone.0051221.s004.tif]
